# Silicosis research priorities for health care, research, and health and safety professionals, and for people exposed to silica in Australia: a research priority setting exercise

**DOI:** 10.5694/mja2.70013

**Published:** 2025-07-29

**Authors:** Hayley Barnes, Sharna Mathieu, Deborah C Glass, Malcolm R Sim, Lin Fritschi, Joanne L Dickinson, Daniel C Chambers, Tim R Driscoll, Graeme Edwards, Nikky LaBranche, Catherine Jones, Jane E Bourke, Ryan F Hoy, Christine R Jenkins, Simon Apte, Anne Holland, Gabriella Tikellis

**Affiliations:** ^1^ Monash Centre for Occupational and Environmental Health Monash University Melbourne VIC; ^2^ Monash University Melbourne VIC; ^3^ Alfred Health Melbourne VIC; ^4^ Lung Foundation Australia Brisbane QLD; ^5^ Griffith University Brisbane QLD; ^6^ Curtin University Perth WA; ^7^ Menzies Research Institute Tasmania University of Tasmania Hobart TAS; ^8^ The University of Queensland Brisbane QLD; ^9^ The Prince Charles Hospital Brisbane QLD; ^10^ The University of Sydney Sydney NSW; ^11^ Royal Australasian College of Physicians Sydney NSW; ^12^ Work and Health Risk Management Brisbane QLD; ^13^ Sustainable Minerals Institute the University of Queensland Brisbane QLD; ^14^ Biomedicine Discovery Institute Monash University Melbourne VIC; ^15^ The George Institute for Global Health Sydney NSW

**Keywords:** Occupational diseases, Lung disease, interstitial

## Abstract

**Objectives:**

To identify the silicosis research priorities of people living with silicosis, workers at risk of silicosis, their partners and caregivers, and of health professionals and researchers.

**Study design:**

Research priority setting exercise; modified James Lind Alliance framework for research priority setting partnerships, comprising an online survey followed by two forums in which thematic analysis and nominal group analysis were used to establish a list of research priorities.

**Setting, participants:**

People with or at risk of silicosis, their partners or caregivers (survey, online forum) and health care professionals, researchers, health and safety professionals (survey, in‐person forum), recruited 14 April – 19 December 2023.

**Main outcome measures:**

Research priorities in four pre‐identified areas: prevention, screening and diagnosis, treatment, and living with and managing the impact of silicosis.

**Results:**

A total of 164 survey respondents (105 medical or research professionals, 34 workers currently or formerly at risk of silicosis, eleven people with confirmed silicosis, and fourteen partners or caregivers) identified 47 key research topics. Fifty‐three health care professionals and thirteen people with or at risk of silicosis and their caregivers then ranked the research topics and developed research questions at the two forums. The highest ranked research priorities were research into assessment and optimisation of the hierarchy of controls, compliance and regulation, establishing minimum standards and developing innovative screening methods, early diagnosis, development of effective treatments, identification of biomarkers for risk of progression, developing an optimal care model that includes mental health care, and estimating the economic impact of silicosis. Both participant groups agreed that research into workplace controls is important, as is improving education and awareness, compliance with preventive measures, and screening and diagnosis, including nationally consistent screening and diagnosis practices. The professional participants rated research into silicosis pathogenesis and biomarkers and technological considerations higher than workers and their carers, who focused more on the barriers for and attitudes of workers, specific treatments, and managing symptoms.

**Conclusions:**

Research into eliminating exposure to silica, early diagnosis of silicosis, preventing disease progression, and reducing the impact of disease were the top research priorities for people with professional or personal interests in silicosis. Our findings should guide research directions and inform policy development.

**The known**: The incidence of silicosis in young workers in Australia is rising. Improving knowledge of how silicosis can be prevented and managed is urgently needed for research, funding, and policy decisions.

**The new**: Both people at risk of silicosis and those involved in their care or silicosis research regard eliminating exposure to silica, early diagnosis and better treatments, and reducing the personal and social impact of silicosis as priorities.

**The implications**: We have identified the research priorities of people directly affected by silicosis or involved in its prevention and management in Australia. They should guide future research, funding, and policy decisions.

Silicosis is a debilitating and potentially fatal parenchymal lung disease caused by the inhalation of respirable crystalline silica dust. There are no effective treatments,^1^, ^2^ and people with silicosis experience reduced quality of life, reduced exercise tolerance, chest pain, impaired mental health, inability to work, and financial stress, and it also has an impact on their families and caregivers.^3^, ^4^ Exposure to silica dust at work can also lead to chronic obstructive pulmonary disease,^5^, ^6^, ^7^, ^8^ autoimmune conditions,^9^, ^10^, ^11^, ^12^, ^13^ an increased risk of lung cancer,^14^ and respiratory infections.^15^

The incidence of silicosis has markedly increased in Australia over the past decade, and is particularly associated with the processing of engineered stone products in the stone benchtop industry.^4^, ^16^ The incidence of acute and accelerated silicosis (development of disease during a relatively short exposure interval) has also increased.^16^ Despite the ban on engineered stone in Australia since July 2024,^17^ many previously exposed benchtop workers are still at risk of developing silicosis, as are workers in other industries, such as construction, mining, quarrying, manufacturing, and tunnelling.^18^

There is an urgent need to improve the prevention and management of silicosis. Following the recommendations of the National Dust Disease Taskforce in 2021,^4^ the Australian government has recognised the importance of enhancing silicosis research. Research priorities must be defined and synthesised to facilitate a coordinated and strategic approach to reducing the impact of silicosis. The prevention and management of occupational lung disease requires collaboration by a wide variety of people in health care, workplace health and safety, industry, trade unions, and governments. In addition, research should be guided by the priorities and needs of the people living with or at risk of the disease and of their partners, caregivers, and families. As it is unclear what matters most to people affected by silicosis, the Australian government commissioned the Lung Foundation Australia to undertake a research priority setting project using the James Lind Alliance framework, which aims to develop a research agenda that is of direct relevance to those affected by a problem and will deliver meaningful outcomes.^19^ This approach has been used to establish research priorities for pulmonary fibrosis,^20^ chronic obstructive pulmonary disease,^21^ mesothelioma,^22^ and asthma.^23^

The aim of our study was to define research priorities in four areas — prevention, screening and diagnosis, treatment, and living with and managing the impacts of silicosis — according to the priorities of people living with silicosis, their partners and caregivers, workers at risk of silicosis, and health professionals and researchers.

## Methods

We undertook a research priority setting exercise, based on the James Lind Alliance framework for research priority setting partnerships,^19^ during 14 April – 19 December 2023. We report our study according to the REPRISE guidelines^24^ (Supporting Information).

### Participants

Two broad groups of people were eligible for participation in the exercise:

- workers at risk of silicosis because they had previously been or were currently exposed to silica dust; people with self‐reported diagnoses of silicosis or silica‐associated conditions; and partners or caregivers involved in the care of a person with silicosis; and
- health care providers, including respiratory and occupational medicine clinicians, nurses, allied health professionals; researchers involved in silicosis research, including exposure science, toxicology, occupational epidemiology, public health, implementation science, basic science, clinical science; and occupational health and safety professionals, including occupational hygienists, regulators, compensators, and union, industry, and legal representatives.

### Study design

The research priority setting exercise comprised four stages: establishing the occupational lung disease network steering committee; identifying research topics in four research areas (prevention, screening and diagnosis, treatment, living and managing the impact of silicosis); reaching consensus about the top research topics in each research area to define research priorities; and synthesising research questions and ranking top research questions in each research area (Box 1).

We modified the James Lind Alliance approach to ensure coverage of broad research areas by focusing on research priorities, rather than selecting the top ten research questions. The aim of this choice was to encourage future support for a wide range of research into the primary, secondary, and tertiary prevention of silicosis.

Box 1 **Silicosis research priority setting exercise: overview**

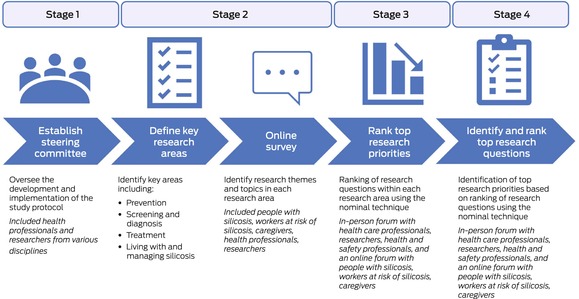

#### Stage 1. Establishing the occupational lung disease network steering committee

Interested health professionals and researchers with expertise in the prevention, diagnosis, or treatment of silicosis were invited by Lung Foundation Australia to participate in the committee (Supporting Information, table 1). The Occupational Lung Disease Network steering committee, established on 14 April 2023, included seventeen people from a wide variety of disciplines (respiratory medicine, occupational and environmental health, radiology, genetics, basic science, qualitative research). The steering committee oversaw the development, refinement, and implementation of the project protocol.

#### Stage 2. Identifying research topics in four pre‐determined research areas

All participants were invited to complete an online survey, after providing active consent. They were recruited via Lung Foundation Australia professional and other mailing lists, study information flyers in occupational lung disease clinics, social media posts, and promotional material sent by email to key organisations and peak bodies. The online survey (Qualtrics; open for seven weeks: 1 July – 15 September 2023) included the open‐ended question: “What are the most important issues or topics you believe researchers should be working [on?]” in each of the four research areas: preventing silica dust exposure, screening or diagnosis of silicosis, treatment of silicosis, and living with silicosis (Supporting Information, table 2). Responses were coded as research themes by three post‐doctoral researchers (authors GT, SM, HB) with experience in qualitative and quantitative research methods.

#### Stages 3 and 4. Defining research priorities by consensus; synthesis and ranking of top research questions

Two research forums were held to present, prioritise, and discuss research topics and specific research questions, based on the survey responses in stage 2. Participants were recruited to attend the forums by advertisements in the online survey and through professional and public interest networks, such as Lung Foundation Australia (https://lungfoundation.com.au). Consent was implied by attendance at the forums.

Health care professionals, researchers, and occupational health and safety professionals were invited to participate in an in‐person forum, which involved small group discussions, each led by a member of the steering committee; the overall process was guided by an independent facilitator experienced in consumer‐based priority setting. Participants were provided with a summary of the online survey results prior to the forum. Each small group discussed these results and then ranked the five most important research priorities in each research area (using Slido, https://www.slido.com); the voting results were then presented to the entire group for discussion and feedback. A second round of small group discussions developed specific research questions for the top three to five priorities in each of the four areas, and then ranked the top three research questions. The results from each group were then reported to the entire group, and the results were analysed using a modified anonymous nominal technique^25^ (using Slido).

Workers at risk of silicosis, people with silicosis, and partners or caregivers were invited to participate in an online forum (Zoom), facilitated by the same independent facilitator and recorded for further analysis. Participants were presented with the research priorities generated at the in‐person forum, which they discussed in groups. Participants were then asked to rank the priorities (using Slido), and then discussed their relevance and importance.

### Ethics approval

The project was approved by the Monash University Human Research Ethics Committee (project 39219).

## Results

A total of 164 respondents completed the online survey (stage 2), of whom 105 were medical or research professionals, 34 were workers currently or formerly at risk of silicosis, eleven had confirmed silicosis, and fourteen were partners or caregivers (Supporting Information, table 3). A total of 47 topics were extracted from the completed questionnaires: fourteen topics in prevention, ten in screening and diagnosis, fifteen in treatment, and eight in living with and managing silicosis (Supporting Information, table 4). In stage 3, research questions were generated and ranked at the in‐person forum and were subsequently presented at the online forum; the online forum participants endorsed the research questions and did not suggest any additions or specific modifications (Box 2).

Box 2 **The top silicosis research questions determined by participants at two post‐survey forums, by research priority area**

**Table:** 

Research priority area	Research priorities	Research questions*
Preventing silica dust exposure and silicosis	Identify and optimise methods of prevention using the hierarchy of controls Evaluate effectiveness of education and awareness campaigns Assess and optimise regulation and compliance Optimise assessment of exposure levels and associated risk Develop exposure monitoring technology Understand barriers to implementing safe work practices	What is the public health return on investment for prevention and compliance activities? What are the barriers and enablers to implementing appropriate controls for preventing silica dust exposure and how are they best addressed? What are innovative solutions for exposure control and decision support related to silicosis prevention? Does engineered stone made from amorphous silica have the same toxicological effects as respirable crystalline silica? How can we improve workplace culture related to risk of developing silicosis? What is the best model of application for real time technology? What are the individual and environmental risk factors for acquiring and developing progressive disease?
Screening and diagnosis	Achieve early diagnosis Establish minimum national screening standards Establish comprehensive national data collection of screening programs and diagnoses Optimise role of lung function and radiology in screening and diagnosis Identify biological indicators of disease Understand and address attitudes of people at risk of silicosis to screening and diagnosis Use advanced technologies in screening and diagnosis Determine optimal screening intervals Understand barriers to screening compliance	What is the best model of screening practice (including risk prediction, screening intervals, cost‐effectiveness, reporting, threshold for high‐resolution computed tomography, other)? What are optimal screening intervals? What are the approaches to and benefits of early testing and diagnosis? What are the biological indicators of exposure and risk to silica dust, including the development of novel technologies? How can occupational respiratory screening be harmonised with the current national lung cancer screening roll‐out? What is the cost effectiveness of prevention, screening, and early diagnosis (both societal and health system)? How can we optimise national data collection on exposure, screening, diagnosis? How can we identify novel diagnostic and prognostic biomarkers of silica‐related disease?
Treatment	Identify indicators of disease progression Understand the impact of comorbid conditions (including mental health, autoimmune conditions, respiratory conditions) Treatment innovations Understand pathogenesis and biomarkers Accessibility of treatment options Assess efficacy of whole lung lavage Identify and address symptom burden Assess efficacy of new or repurposed medications	What is the pathogenesis of silicosis? What is the potential for new antifibrotics? What biomarkers (including genomics) should be used for screening, early diagnosis, risk of progression and develop treatment pathways? What are the best experimental models to recapitulate silicosis in humans and test therapies? What are the most effective measures of impairment and endpoints for treatment, including patient‐centred outcomes? What does an optimal model of care for silicosis involve?
Living with and managing the effects of silicosis	Managing health and well‐being Working after a diagnosis of silicosis Improve care co‐ordination Optimise compensation assessment and processes Development and access to support services Symptom management Assess the financial and economic impact of silicosis and silica‐related diseases	What factors impact/influence mental health determinants and how are they related? What are the barriers and levers for change in improving the pathway and outcomes to compensation? What is the best holistic/integrated/multidisciplinary optimal care model that is strongly linked to the individual care journey (not just medical model)?

* A more comprehensive list of research questions is included in the Supporting Information, table 5.

### Demographic characteristics of forum participants

The in‐person forum (stage 3) was attended by 53 participants: eighteen academics (interest areas included occupational hygiene, pharmacology, epidemiology, basic science, allied health), sixteen health care professionals, six legal professionals, four regulators, three people from non‐governmental organisations, two occupational hygienists, two union representatives, and two industry representatives. The participants were purposively allocated to six small groups to achieve similar numbers of academics, health care professionals, legal professionals, and others in each group. The online forum was attended by thirteen people: eight with silicosis, one partner or caregiver, and five workers or community members at risk of silicosis. As the online survey was completed anonymously, it could not be determined whether forum participants had completed the survey. The top research priorities by participant group are shown in Box 3, Box 4, and Box 5; the top priorities for all forum participants are ranked in Box 6.

Box 3 Ranking of research priorities by research area: results of the in‐person forum for 53 health care professionals, researchers, and occupational health and safety professionals*

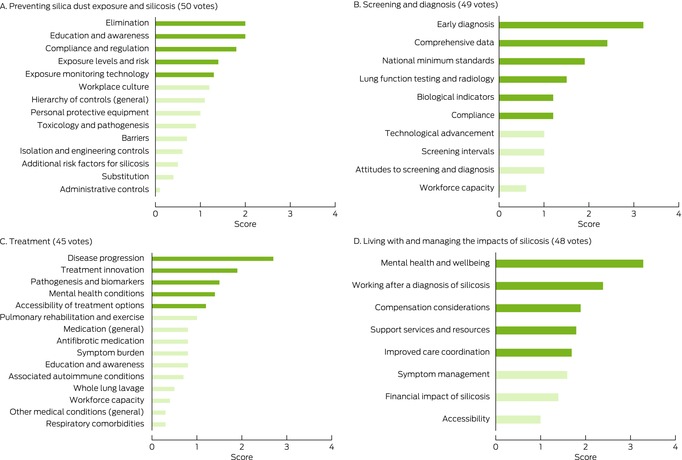

* For derivation of ranking poll scores, see reference 26.

Box 4 Ranking of research priorities by research area: results of the online forum for thirteen people with silicosis, workers at risk of silicosis, and partners or caregivers*

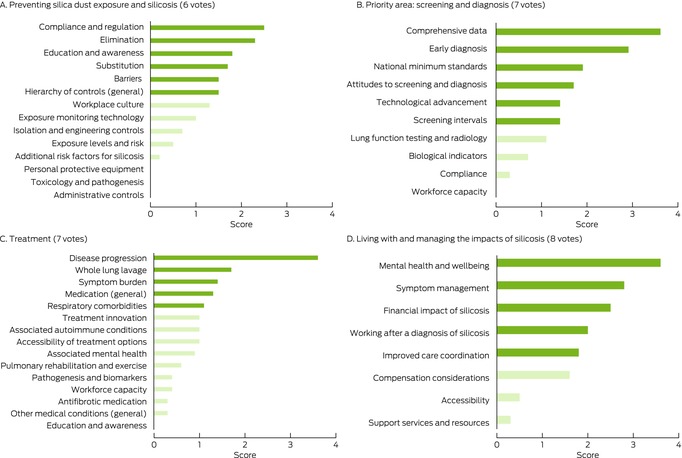

* For derivation of ranking poll scores, see reference 26.

Box 5 **Ranking of research priorities by research area: comparison of the two forum participant groups**

**Table:** 

Outcome	Preventing silica exposure and silicosis	Screening and diagnosis	Treatment	Living with silicosis
Consensus priorities	Identify and optimise methods of prevention using the hierarchy of controls Evaluate effectiveness of education and awareness campaigns Assess and optimise regulation and compliance	Achieve early diagnosis Establish minimum national screening standards Establish comprehensive national data collection of screening programs and diagnoses	Identify indicators of disease progression Understand the impact of comorbid conditions (including mental health, autoimmune conditions)	Managing health and wellbeing Working after a diagnosis of silicosis Improve care co‐ordination
Higher priority for health care professionals, researchers, and occupational health and safety professionals	Optimise assessment of exposure levels and associated risk Develop exposure monitoring technology	Optimise role of lung function and radiology in screening and diagnosis Identify biological indicators of disease Understand barriers to screening compliance	Accessibility of treatment options Understand pathogenesis and biomarkers Treatment innovations	Development and access to support services Optimise compensation assessment and processes
Higher priority for people with silicosis, workers at risk of silicosis, and partners or caregivers	Understand barriers to implementing safe work practices	Understand and address workers’ attitudes to screening and diagnosis Use advanced technologies in screening and diagnosis Determine optimal screening intervals	Assess efficacy of whole lung lavage Identify and address symptom burden Assess efficacy of new or repurposed medications	Symptom management Assess the financial and economic impact of silicosis and silica‐related diseases

Box 6 **Overall ranking of silicosis research priorities by all exercise participants**

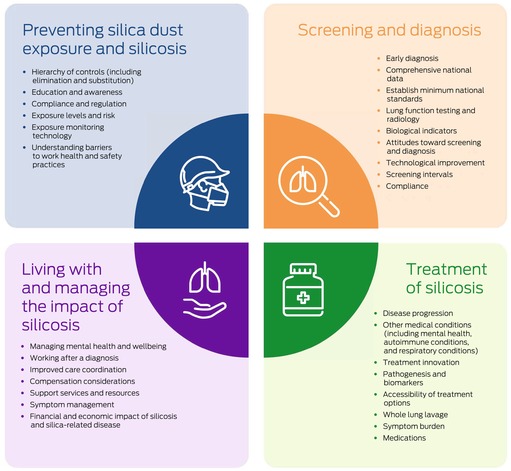

### Preventing silica dust exposure and silicosis

Eliminating exposure was ranked by all participants as the highest research priority, but health care professionals noted that research in all components of the hierarchy of controls (elimination, substitution, isolation, engineering controls, personal protective equipment) were necessary for preventing silica dust exposure. Specific research questions included assessing the safety of new substituted materials and real‐time dust monitoring devices.

Identifying barriers to and enablers of effective compliance with the hierarchy of controls (for identifying and ranking safeguards for protecting workers from hazards, including elimination, substitution, engineering controls, administrative controls, personal protective equipment) was identified as an important research priority. Overcoming barriers specific to rural areas and for people from culturally and linguistically diverse backgrounds was also discussed.

Health care professionals acknowledged that research in implementation science (adopting what is known into policy and practice) is essential, including into increasing responsibility and accountability at all levels (government, employers, regulators, workers); active worker participation is central to averting silica‐related diseases. Similarly, the online forum participants reported that active participation by workers in qualitative patient‐centred research empowers them to speak up about health and safety matters, and that widespread cultural change was needed to reduce the stigma attached to speaking up in the workplace. Health care professionals ranked investigation of the best methods and the implementation of hierarchy of controls as a higher research priority than did workers and community members, who ranked education and overcoming barriers and changing workplace culture as priority research areas.

### Screening and diagnosis

Early diagnosis was a high priority research area for all participant groups. They regarded national consistency in screening programs and diagnostic practices as important and saw potential benefits in a best model of screening practice for all workers formerly or currently exposed to silica dust. It was agreed that research into screening methods and screening intervals was required, and that any screening models should undergo a cost‐effectiveness analysis that compared the cost of screening with the numbers of lives, disability‐adjusted life‐years, and quality‐adjusted life‐years saved.

All participant groups also discussed the invasiveness of current screening methods and whether future research could develop less invasive alternatives. They also highlighted the benefit of a central registry or repository with minimum data standards that could benefit both researchers and workers, who noted that information can be lost when moving house or between workplaces. Workers and community members were somewhat reluctant to undergo screening because they feared receiving bad news (being diagnosed with silicosis), suggesting that any research into and implementation of a screening program and central registry should involve input by workers and community members.

### Treatment of silicosis

People with silicosis described the “torture” of not being able to breathe and the importance of developing suitable treatment options for silicosis that are available in a timely and equitable manner across Australia. Health care professionals discussed the lack of effective treatments and how treatment innovation was required. Top research questions in this area included identifying biomarkers (including genetic markers) for screening, early diagnosis, estimating the risk of progression, and determining treatment; identifying relevant outcome measures of impairment and endpoints for treatment, including patient‐centred outcomes; and identifying the best experimental models of silicosis in humans, including models for testing therapies. Research priorities for worker and community member participants were reducing the symptom burden; that is the need for developing treatments that are directed at the pathogenesis of the disease, but also treatments that reduce symptom severity.

### Living with and managing the impact of silicosis

Both health care professionals and workers and community members ranked research into maintaining mental health and wellbeing as the top priority in this area. Top research questions included identifying factors that influence mental health and how they can be modified, and developing an optimal multidisciplinary care model that is more strongly linked with the individual's care journey than with a medical model. Both groups also agreed that further research into the economic impact of disease is needed, both for the individual worker (including compensation) and on the government and societal levels. The development of a more holistic optimal care model based on evidence and the needs of the individual was a top priority.

## Discussion

In our structured research priority setting exercise, we identified the top silicosis research priorities for health care professionals, researchers, and occupational health, safety professionals and for people with silicosis, at risk of silicosis, and partners or caregivers in four areas: preventing silica dust exposure and silicosis, screening and diagnosis, treatment, and living with and managing the impact of silicosis. Research into the hierarchy of controls, early diagnosis, treatments for preventing disease progression, and developing an optimal care model that includes mental health care were ranked as most important.

We used a structured approach for our research priority setting project, a modification of the James Lind Alliance method. We engaged with a wide variety of people professionally or personally concerned with silicosis to identify occupational disease research priorities. In each research area, the agreement between people with or at risk of silicosis and their caregivers and professionals from various disciplines about research priorities was reasonable. Both participant groups agreed that research into workplace controls and their implementation is important, as is improving education and awareness, compliance with preventive measures, and screening and diagnosis; they also agreed that earlier and nationally consistent screening and diagnosis practices were needed, and more knowledge about disease progression and the influence of other medical conditions, managing mental health, transitioning to new work, and improving care coordination.

However, some clear differences are important. The professional participants rated research into silicosis pathogenesis and biomarkers and technological considerations more highly than workers and their carers, who focused more on the barriers for and attitudes of workers, specific treatments, and managing symptoms as equally valuable for directing future research.

Our modified James Lind Alliance approach aimed to identify research priorities in four broad research areas to encourage future support for a broad scope of research into the primary, secondary, and tertiary prevention of silicosis, a rapidly evolving area. Our research setting project was conducted before the Australian government announced its ban on engineered stone products, and research priorities may change as policy changes. However, our deliberately broad and inclusive scope allows for evolution of both policy and knowledge regarding silicosis, and could inform research priorities in countries where engineered stone is still used. Even with the ban, many workers already exposed to engineered stone products will still require screening and diagnosis, and a proportion will unfortunately develop silicosis and require treatment and support with living with the disease. Substitution with materials not including silica needs to be investigated. Further, there are many other occupational sources of respirable crystalline silica, including in construction and mining, where research should be focused.

One component of the James Lind Alliance approach is uncertainty verification; that is, determining whether an uncertainty has already been investigated in earlier research. The James Lind Alliance suggests examining each question using high level evidence from systematic reviews, guidelines, and large scale registry databases.^19^ However, as silicosis research, particularly that related to engineered stone, is relatively recent, high level evidence is unlikely to be available. As part of our protocol, we plan to examine the impact of our research priority setting project in 2030, when the evidence base should be more substantial.

During the research priority setting project, we noted a distinction between the need for research to answer specific questions (for example: How can novel diagnostic and prognostic biomarkers of silica‐related disease be identified?) and research into how to best translate evidence into practice. Implementation research requires the involvement of not only researchers, but also of governments, policy makers, regulators, and workers, as was noted in the forums. It is hoped that the generation of research priorities will not only assist researchers, but also others involved in improving the health and safety of people working with silica and those with silicosis.

### Limitations

Our study involved a wide variety of people in Australia with an interest in silicosis to identify research priorities, conducted with a systematic approach. The recruitment process for our online survey and forums precludes any conclusions about the representativeness of our participants. The online forum avoided the need for travel, enabling the participation of people with or at risk of silicosis in different locations. However, the inherent limitations of virtual discussions, compared with in‐person discussions, cannot be overlooked. The relatively small number of people at risk of or living with silicosis involved in the study, and none from culturally and linguistically diverse backgrounds, who comprise a substantial proportion of stone benchtop workers,^16^ were also limitations. Future research should actively involve workers and other community members in its design and implementation.

### Conclusion

In a silicosis research priority exercise, we found that research into the elimination of exposure and other hierarchy of controls of silica use, early diagnosis, treatment for preventing disease progression, and the development of an optimal care model, including mental health care, were the top research priorities for people with either professional or personal interest in silicosis. Our findings could guide future silicosis research and policy development.

## Open access

Open access publishing facilitated by Monash University, as part of the Wiley – Monash University agreement via the Council of Australian University Librarians.

## Competing interests

Anne Holland is the President of the Thoracic Society of Australia and New Zealand.

## Data sharing

The de‐identified data we analysed are not publicly available, but requests to the corresponding author for the data will be considered on a case‐by‐case basis.

## Supporting information

Supplementary methods and results
