# Supplementary material for: Silicosis research priorities for health care, research, and health and safety professionals, and for people exposed to silica in Australia: a research priority setting exercise
Source: Med J Aust. 2025 Jul 29;223(5):257–64. doi: 10.5694/mja2.70013 (PMC12399050; doi:10.5694/mja2.70013)
Supplement: Supplementary file 1 — Supplementary methods and results [file MJA2-223-257-s001.pdf]

The Medical Journal of Australia

Supporting Information

Supplementary methods and results

This appendix was part of the submitted manuscript and has been peer reviewed.
It is posted as supplied by the authors.

Appendix to: Barnes H, Mathieu S, Glass DC, et al. Silicosis research priorities for health care, research, and health and safety professionals, and for people exposed to silica in Australia: a research priority setting exercise. *Med J Aust* 2025; doi: 10.5694/mja2.70013.

Supplementary methods

Table 1. Occupational Lung Disease Network Steering Committee Members

Chair – Dr Hayley Barnes PhD FRACP MPH MBBS BBioMedSci(Hons) - Monash University

Deputy Chair – Professor Catherine Jones BSc MBBS FRCR FRANZCR GAICD - University of Sydney

Dr Simon Apte PhD - University of Queensland

Associate Professor Jane Bourke PhD BSc(Hons) - Monash University

Professor Daniel Chambers MD FRACP FRCP MBBS - University of Queensland

Professor Jo Dickinson PhD BSc(Hons) - University of Tasmania

Professor Tim Driscoll BSc(Med), MBBS MOHS PhD FAFOEM FAFPHM - University of Sydney

Dr Graeme Edwards FAFOEM - Australasian Faculty of Occupational and Environmental Medicine

Emeritus Professor Lin Fritschi PhD MBBS, FAPHM- Curtin University

Professor Deborah Glass PhD - Monash University

Professor Anne Holland PhD BAppSc - Monash University

Dr Ryan Hoy FRACP MOEH MBBS - Monash University

Professor Christine Jenkins AM MD FRACP FThor Soc FAAHMS - University of Sydney

Ms Nikky LaBranche BSc MBA - University of Queensland

Professor Lidia Morawska PhD MSc – Queensland Institute of Technology

Emeritus Professor Malcolm Sim AM, MBBS, MSc, PhD, FAFOEM - Monash University

Dr Gabriella Tikellis PhD - Monash University

Lung Foundation Australia Members

Sharna Mathieu

Elizabeth Early

Mark Brooke

Table 2. Online survey

Research Priority Setting in Occupational Lung Disease

Project ID number 39219

Project Sponsor: Lung Foundation Australia and Monash University

Chief Investigator/s:

Hayley Barnes
Department of Planetary Health
Email: Hayley.Barnes@monash.edu
Sharna Mathieu
Lung Foundation Australia
Email: sharnam@lungfoundation.com.au

You are invited to take part in this study. Please read this Explanatory Statement in full before deciding whether to participate. If you would like further information regarding any aspect of this project, you are encouraged to contact the researchers via the email addresses listed above.

The purpose of this online survey is to identify research needs in silicosis. Specifically, results from this survey will be used to identify where researchers, governments, and other funding bodies should be directing their attention, time, and resources.

This work is being conducted by Lung Foundation Australia, in partnership with multidisciplinary researchers from across the country, and Monash University. Lung Foundation Australia was funded by the Department of Health and Aged Care to complete this work and will present the outcomes to the Department in an annual report.

All responses are welcome, and we encourage you to list as many research questions or comments as you can. Responses are anonymous. In the event you do choose to leave your contact details, this will be kept confidential, and all responses will be de-identified.

Which of the following best describes you?

- ☐ I am someone living with silicosis
- ☐ I am a worker exposed to silica dust but do not have silicosis (e.g., construction, mining, tunnelling)
- ☐ I am someone undergoing investigation and suspect I have silicosis I am a family member or caregiver of someone living with silicosis
- ☐ I am someone working to help people living with silicosis or workers at risk of silicosis

If 'someone living with silicosis' or 'worker' is selected

Which industry have you mostly worked in?

- ☐ Construction
- ☐ Mining and quarrying
- ☐ Manufacturing
- ☐ Tunnelling
- ☐ Stone benchtop fabrication
- ☐ Other ____________________

If 'someone working to help people living with silicosis or at risk of silicosis is selected'

Which of the following best describes your professional role? [please select all that apply]

- ☐ Researcher / Academic (main area of interest) ________________
- ☐ Epidemiologist (main area of interest)______________
- ☐ Respiratory / Respiratory and Sleep Physician
- ☐ Occupational and Environmental Physician
- ☐ General Practitioner
- ☐ Radiologist
- ☐ Psychiatrist
- ☐ Rheumatologist
- ☐ Occupational Hygienist
- ☐ Occupational Health and Safety Professional Respiratory Care Nurse
- ☐ Occupational Health Nurse Lung Cancer Nurse
- ☐ Nurse (other) ________________________
- ☐ Radiographer
- ☐ Physiotherapist
- ☐ Exercise Physiologist Occupational Therapist
- ☐ Rehabilitation Counsellor
- ☐ Psychologist
- ☐ Social Worker
- ☐ Public Health / Health Promotion
- ☐ Union / Worker representative
- ☐ Industry representative
- ☐ Solicitor / Lawyer
- ☐ Workplace safety regulator
- ☐ Other ____________________

How long have you been working with silicosis patients or at-risk workers, in occupational health and safety and/or involved in topics related to occupational lung health?

- ☐ Up to 2 years
- ☐ 2-4 years
- ☐ 5-7 years
- ☐ 8-10 years
- ☐ More than 10 years up to 20 years
- ☐ More than 20 years

For all respondents to complete onwards

What is your gender identity?

- ☐ Male
- ☐ Female
- ☐ Non-binary / third gender
- ☐ Prefer not to say

Do you identify as Aboriginal and/or Torres Strait Islander?

- ☐ No
- ☐ Yes, Aboriginal
- ☐ Yes, Torres Strait Islander
- ☐ Yes, both Aboriginal and Torres Strait Islander
- ☐ Prefer not to say

What is your age?

- ☐ 18-24
- ☐ 25-34
- ☐ 35-44
- ☐ 45-54
- ☐ 55-64
- ☐ 65-74
- ☐ 75-84
- ☐ 85 +

Which state or territory are you primarily based in?

ACT NSW NT QLD SA TAS VIC WA

What language do you prefer to speak at home? ___________________________________

ISSUES OR TOPICS THAT ARE IMPORTANT TO YOU

This next section asks you to list any issues or topics about silica dust exposure and/or silicosis you believe researchers should be working on to improve or better understand.

We encourage you to be creative and include any topic areas you believe are important. Some aspects of this survey may be more relevant to your experience than others - this is okay.

Please list as many issues/topics as you can, or feel free to make descriptive comments so we understand the issue. The use of dot-points is encouraged.

What are the most important issues or topics you believe researchers should be working regarding:
PREVENTING SILICA-DUST EXPOSURE?

SCREENING OR DIAGNOSIS OF SILICOSIS?

TREATMENT OF SILICOSIS?

LIVING WITH AND MANAGING THE IMPACT(S) OF SILICOSIS?

Please list any other important issues or topics in your opinion or experience. These can be related to the above areas or can be areas not mentioned above.

Supplementary results

Table 3. Backgrounds of the 164 survey participants

**Background**	**Number**
**Health care professionals***	
Occupational and environmental health physician	17
Occupational hygienist	16
Nurse	13
Respiratory physician	7
Physiotherapist	4
General practitioner	3
Social worker	2
Psychologist	1
Radiologist	1
Occupational therapist	1
Rheumatologist	1
**Academics***	
Researcher/academic not otherwise specified	33
Public health academic/epidemiologist	10
**Health and safety professionals**	
Union representative	4
Lawyer	3
Workplace safety regulator	2
Industry representative	1
Other	7
**At risk /living with silicosis and caregivers**	
Worker at risk of silicosis	34
Partner/caregiver	14
Person living with silicosis	11

* Responses by an individual in both categories possible.

Table 4. Summary of survey responses

Priority Area: Preventing silica dust exposure and/or silicosis

1. Compliance and regulation
• Legislative compliance, enforcement, and penalties
• Audit/monitoring of workplaces to identify preventative controls in place and compliance with exposure standards
• Determining the effectiveness of regulation and compliance with workplace exposure standards, preventative controls, and inspections
• More transparent public information on exposure levels across tasks and industries, workplace illnesses, and compensation agencies to aid worker decision making
• Utilise national data to identify malpractice
• Licensing of demolition workers
• Mandatory and early screening using best-practice methods

2. Hierarchy of controls (general)
• Verify the effectiveness of current preventative controls according to task, occupation, and industry
• Determine minimum standards of exposure control for various tasks or conditions
• Design and improve dust control technology, including real time exposure control
• Improved implementation of the Hierarchy of Controls in workplaces and addressing barriers
• Impact of environmental conditions (e.g., wind) on the effectiveness of various exposure controls
• Exposure control at the source of dust generation
• Determine the current use of exposure controls
• Safe by design practices
• Exposure control for family members with secondary exposure
• Determine the cost-benefit of preventative measures

3. Elimination
• Role, feasibility and (cost) effectiveness of a ban on engineered stone products
• Eliminate unnecessary products/materials that contain high concentrations of silica

4. Substitution
• Identify and determine the actual safety of lower silica content engineered stone products that still contain other harmful compounds
• Investigate viable alternatives to materials containing high concentrations of silica in all industries

5. Isolation and engineering controls
• Automation of processes to limit exposure
• The role and effectiveness of current engineering controls and their failure points
• Investigate alternative fluids for wet processing of silica containing materials
• Impact of a historical lack of sealed and air-conditioned slow-moving vehicles (e.g., transport)
• Sealed devices for safer disposal and/or recycling of processed dust
• Impact of wet processing methods on the environment (use of water)

6. Administrative controls
• Current workplace training and induction practices and level of awareness
• Improve tailored training and induction on the risks of silica and exposure control strategies – particularly for workers with lower formal educational attainment, from culturally and linguistically diverse backgrounds and younger workers (apprentices)
• Develop multilevel workplace education programs

7. Personal Protective Equipment (PPE)
• Improved respiratory protection technology and equipment (including comfort and cost)
• The role, viability and effectiveness of current PPE and their failure points

8. Exposure monitoring technology
• Develop low cost and sensitive airborne/atmospheric exposure monitoring technology, particularly for exposure at low levels and in the field
• Effectiveness of exposure monitoring technology (including silica, silicates and total dust levels)

• Real time exposure monitoring
• Video based exposure monitoring
• On person exposure monitoring
• Use of Artificial Intelligence (AI) technology to process exposure data

9. Exposure levels and risk
• Determine exposure thresholds to better understand the impact of one- off large exposures or repeated, lower-level exposures over a working lifetime
• Determine levels of exposure and likely trajectory of silica related disease if control measures are utilised (laboratory and real-world conditions)
• Database of exposure measurements from all occupations and industries to identify number of exposed workers and overcome a current reliance on reporting exceedances of workplace exposure standards
• Burden of silica exposure on the healthcare system and society
• Determine cooccurring exposures in the processing of engineered stone or other high-risk activities, occupations, or industries
• Determine a health-based workplace exposure standard for respirable crystalline silica (is there a safe level of exposure)?
• Exposure and task-based risk profiles for airborne silica
• Consider particle size and exposure duration
• Monitor silica exposure trends over time and determine whether exposure levels are reducing over time
• Determine workers most at risk of exposure to respirable crystalline silica (by industry, role or task)
• Investigate risk of exposure in the demolition industry (legacy engineered stone products etc.)

10. Additional risk factors for silicosis
• Determine additional and intersecting risk factors for developing silicosis – including possible role of genetics, smoking history, medication history, cultural background etc.
• Determine high risk occupations and industries
• Utilise advances in genetic understanding of lung fibrosis
• Understand role of cooccurring exposures on the development or severity of silicosis

11. Toxicology and pathogenesis
• Toxicological properties and biological effect of other components of engineered stone to determine risk levels and interaction with respirable crystalline silica (including animal toxicology studies)
• Characterise the dust generated by the processing of engineered stone and uptake by the lung (type and size of silica and other components)
• Understand changes to engineered stone over time or under certain conditions (heated, exposure to light)
• Determine risk from the use of amorphous silica used in alternatives to traditional engineered stone with a high crystalline silica content
• Understand the mechanisms leading to lung and autoimmune disease
• Comparison of lung disease between similar silica content natural rock versus engineered stone

12. Education and awareness
• Develop and evaluate targeted public health campaigns about the harms of silica exposure for workers, family members, and the community / general public
• Raise awareness as to the benefits of engaging in health screening/monitoring
• Increased health professional education and awareness
• Educate employers about the cost-benefit safe work practices
• Engage workers to be involved in the tailoring of key messages
• Tailor education and awareness programs to suit different communities, cultures and languages
• Effectiveness of education and awareness campaigns on improving safer work practices
• Consumer awareness of alternative building materials with low or no silica content

13. Workplace culture
• Understand employer/employee attitudes and norms in relation to exposure control in high-risk occupations and industries (typically male- dominated)
• Behavioural insights in workplace safety failures to date
• Improve workplace safety culture to make exposure control a non-negotiable expectation
• Survey of all members of a workplace (employer, employee, management etc.) to encourage multilevel organisational behaviour change
• Involve workers in a bottom-up safety approach

14. Barriers
• Determine barriers to establishing compliance with regulations within workplaces (e.g., productivity concerns)
• Determine barriers to the implementation of Hierarchy of Controls
• Financial barriers to the implementation of health monitoring and/or Hierarchy of Controls in businesses (especially small and medium sized enterprises)
• Cultural and linguistic barriers to engaging with health monitoring and/or implementing the Hierarchy of Controls
• Educational barriers to engaging with health monitoring and/or implementing the Hierarchy of Controls

Priority Area: Screening and diagnosis

15. Early diagnosis
• Reliable screening methods to facilitate early diagnosis of silicosis (and other silica-related disease) and treatment
• Determine clinical relevance and impact of identifying very early-stage simple silicosis
• Manage influx of new patients and system burden due to wider screening

16. Comprehensive national data
• Centralised data collection and analysis (national register)
• Establish national database including history of the worker and exposure origin – facilitate data linkage
• Establish a baseline for all workers across occupations and industries where silica exposure is likely to occur
• Determine silica-related disease trajectories and whether silicosis is always fatal
• Longitudinal data collection and prospective and retrospective cohort designs with capacity for data linkage
• Determine incidence and prevalence of silicosis and other silica-related disease and level of impairment
• Audit of historical screening and/or compensation cases to confirm numbers according to best practice methods (e.g., review of asbestosis or asbestos related pleural plaque disease cases)
• Develop improved predictive models to assist with assigning prognosis and assessing compensation
• Long term burden of disease studies (impact on family, communities, healthcare system, workforce etc.)
• Economic evaluation

17, Establish national minimum standards
• All states and territories following the same processes and eligibility criteria
• Determine best practice for the screening of workers considering cost, efficiency, continuum of care, low risk to workers
• Improved understanding of the effectiveness and implementation of current best practice methods for screening and diagnosis
• Access to national testing and screening resources made more convenient
• Financial cost of screening not to be borne by workers and waitlists/costs of accessing specialists once diagnosed may be prohibitive to workers engaging in screening

18. Screening intervals
• Determine the optimal interval for screening (e.g., annual screening)
• Cost-benefit of more regular screening

19. Lung function testing and radiology
• Compare the effectiveness of high and low dose HRCT versus chest x- ray (ILO standards) across occupations and industry
• Role and validity of spirometry as well as lung volume and diffusing capacity of the lungs for carbon monoxide (DLCO) in screening of workers
• Advanced non-radiation-based imaging (e.g., MRI ventilation inhomogeneity imaging)
• Reduce impact on workers undergoing screening (e.g., less intensive, radiation)

20. Technological advancement
• Develop novel and/or improved techniques for the detection of early lung injury (e.g., increased sensitivity and specificity, more cost efficient)
• Determine how Artificial Intelligence (AI) technology can be utilised in processing images and diagnostic data
• Transportable and/or virtual screening methods, especially for rural and remote workers

21, Biological indicators
• Effectiveness of nasal swabs, sputum samples, and/or exhaled breath in the screening/diagnosis of silica-related disease
• Identify novel diagnostic and prognostic biomarkers of silica-related disease
• Determine whether tissue damage to other organs (e.g., nasal tissue, conjunctivae, skin) can be used as surrogate markers
• Role of angiotensin converting enzyme (ACE)or immunological markers
• Role of genetic testing

22. Attitudes toward screening and diagnosis
• Understand attitudes toward health monitoring and screening (former and current workers, apprentices, managers, professional bodies, unions)
• Determine how to better engage workers and employers in screening and engaging with primary healthcare
• Understand reasons why people do not engage in screening (e.g., financial impact of a diagnosis)

23. Compliance
• Level of compliance with health monitoring being undertaken by employers across high-risk industries for current and former workers
• Those working in industry where they are likely exposed to silica dust attend mandatory screening
• Mandatory monitoring of those with significant exposures

24. Workforce capacity
• Increase education and training for primary healthcare providers on screening for silica-related disease and associated best practice tests
• Increase number of radiologists with necessary qualifications and skills in imaging of silica-related disease
• Greater allocation of funding and resources to reduce waitlists and improve access in rural and remote areas
• Timely diagnosis and results
• More supportive wraparound care for anyone undergoing investigation
and/or diagnosis of silicosis

Priority Area: Treatment of silicosis

25. Medications (general)
• Define and evaluate current practice and whether commonly prescribed medications affect silicosis
• Role of inhaler medications
• Impact of current practice on patients and their families in lieu of an established cure (i.e., number of medications prescribed, cost)
• Address side-effects

26. Symptom burden
• Determine rate and pattern(s) of disease progression (e.g., why do some patients with silicosis have well preserved lung function and minimal radiological disease yet report/experience severe symptoms?)

27. Disease progression
• Treatment to improve lung function over time
• Slow or reverse silicosis disease progression and fibrosis
• Reverse effects of silica exposure on the body
• Destabilise cytokines

28. Antifibrotic medication
• More clinical trials, particularly to be able to rapidly repurpose existing antifibrotic treatments
• Effectiveness of antifibrotic medications used early
• Current prescription/use of antifibrotics and effectiveness

29. Pulmonary rehabilitation and exercise
• Effectiveness of pulmonary rehabilitation and exercise for people diagnosed with silicosis (functional improvement, shortness of breath on exertion, stamina, weakness, effort tolerance)
• Increased accessibility of pulmonary rehabilitation

30. Whole lung lavage

• Role and efficacy of whole lung lavage in treating or controlling the rate of fibrosis after silica exposure
• Determine whether whole lung lavage improves functional impairment
• Improve invasiveness of the procedure
• Develop equipment and use of artificial intelligence (AI) technology in lavage procedures
• Expand clinical trials

31. Treatment innovation
• Develop and test new curative treatments, particularly for progressive massive fibrosis and to avoid lung transplantation
• Adopt precision therapy approaches for idiopathic pulmonary fibrosis for silicosis patients
• Utilise lung tissue / biobanks
• Allogeneic bone marrow transplantation for moderate disease and/or autoimmune manifestations
• Investigate immunosuppressants / biologics / monoclonal antibodies
• Lung regenerative medicine technologies
• Prophylactics

32. Pathogenesis and biomarkers
• Better understand disease pathogenesis and molecular mechanisms driving silicosis and progressive massive fibrosis
• Develop biomarkers to identify new therapeutic targets

33. Associated autoimmune conditions
• Wider screening and education on scleroderma and immune related disorders associated with silicosis for all respiratory physicians and respiratory health clinics
• Impact of treating associated immune conditions versus silicosis itself
• Improve fragmented diagnosis and care for silicosis and related autoimmune conditions (e.g., scleroderma)

34. Associated mental health conditions
• Treatment for associated mental health problems
• Determine impact of cooccurring mental health and/or substance use on treatment options and effectiveness

35. Other respiratory conditions
• Determine whether silicosis exacerbates other respiratory conditions
• Role of treatment in recurrent chest infections / pneumonia
• Synergy between treatment for silicosis and other occupational lung disease and/or chronic obstructive pulmonary disease (COPD)

36. Other medical conditions (general)
• Identify and treat other associated medical conditions related to silica exposure and/or silicosis

37. Workforce capacity
• Improved systems and communication between healthcare team
• Multidisciplinary team management
• Increased funding and resources
• Greater education and awareness among healthcare providers to enhance skill and capacity in silicosis and related conditions
• Promote awareness and upskilling of general practitioners of whom to refer to and when
• Enhance workforce capacity

38. Accessibility of treatment options
• Enhance rural and remote services and virtual/mobile healthcare infrastructure
• Understand and overcome cost related barriers to accessing treatment within the context of inability to work/compensation
• More comprehensive general health assessments of working age people (in certain occupations and industries)
• Equity considerations for those who are most risk of developing silicosis

39. Education and awareness
• Address worker and family misconceptions about treatments and prognosis
• Patient education and communication

• Promote balance in worker attitudes between taking prevention seriously and not relying on treatment developments (e.g., whole lung lavage)
• Increase health literacy

Priority Area: Living with and managing the impact of silicosis

40. Mental health and wellbeing
• Understand the impact of silicosis on the mental health and wellbeing of patients and families (including offspring)
• Impact of a diagnosis on mental health (psychological injury) and immediate support requirements
• Ask workers, patients, family members, and the community about their needs and the impact of silicosis
• Transparent communication and patient education
• Future / family planning and impact of silicosis on different life stages (raising a family, retirement)
• Determine the impact and support needs of coworkers / employers regarding mental health and wellbeing when a colleague is diagnosed

41. Financial impact of silicosis
• Financial stress as a result of a silicosis diagnosis
• Financial support
• Working age impacts e.g., childcare support

42. Symptom management
• Symptom self-management education
• Coping strategies
• Diet and lifestyle strategies
• Monitor disease progression and symptoms

43. Working after a diagnosis of silicosis
• Strategies and adjustments to remain in chosen profession for as long as the person wishes
• Appropriate and meaningful work alternatives
• Impact of continued possibility of exposure on disease versus being forced to leave their chosen profession
• Retraining and employability options for those who have functional work years ahead of them
• Identify barriers and enablers for early return to meaningful work
• Clinical guidelines regarding continued work relative to lung function and stage of disease
• Effectiveness of return-to-work programs and vocational support
• Determine how best to support workers transitioning back into the workforce or new roles
• Discrimination and negative attitudes once returning to previous role or workplace
• Suitable information about work prospects and vocational support / pathways (e.g., culturally and linguistically diverse communities, education)
• Understand the impact of continued work versus stopping work on mental health and wellbeing
• Impact of return to work in chose profession on compensation
• Cost-benefit of recommendations to stop work

44. Compensation considerations resources
• Determine barriers and enablers to early decision on compensation claims for silicosis and associated conditions
• Best practice for compensation systems
• Family support and compensation
• Determine the benefit of compensation to people with silicosis and their families
• Understand the impact of being involved in compensation claims and legal proceedings on health and wellbeing
• Extend time limits of compensation for the increasing number of people diagnosed with silicosis through increased routine screening without any symptoms or functional impairment
• More comprehensive compensation akin to other occupational lung disease (e.g., mesothelioma)
• Role of additional social welfare

45. Improved care coordination and delivery
• Better access to coordinated multidisciplinary support services including allied health and pulmonary rehabilitation
• Increased community respiratory teams and home visits

• More dedicated nursing staff and equipment
• Upskilling health professionals in bedside manner and patient education
• Coordinated and systematic approach for an integrated patient experience with transferrable records
• Rapid translation of evidence-based care strategies

46. Accessibility
• Identify current access and determine needs
• Services for poorly resourced areas and key risk groups
• Culturally and linguistically diverse communities and patients

47. Support services and resources
• Effective peer support programs or groups
• Support for family members and caregivers, including offspring
• Understand long term support needs and identify gaps/needs
• Determine how to best engage people in available support services and resources

Table 5. Research questions by priority area

Priority Area: Prevention

Top Research Questions

- What is the public health return on investment for prevention and compliance activities? -Core industries – quarrying, construction, mining, demolition
- What are the barriers and enablers to implementing appropriate controls for preventing silica dust exposure and how are they best addressed (implementation science)?
- Identify and assess innovative solutions for exposure control and decision support related to silicosis prevention
- Does engineered stone made from amorphous silica have the same toxicological effects as respirable crystalline silica?
- What are the barriers and enablers to having effective compliance? Including in remote, rural and culturally and linguistically diverse communities
- What is the best model of application for real time technology
- How can we improve workplace culture:
  - Related to risk of developing silicosis
  - How can you improve a culture of workplace health and safety
  - How can we motivate small businesses
- Material science – what is the pathogenesis of new/emerging silica containing materials/products
- What are the health impacts of substitution products?
- What are individual and environmental risk factors for acquiring and developing progressive disease?
  - Demographics
  - Dust characteristics
  - Individual characteristics
- Can we diagnose (pre-silicosis) silicosis risk using minimally invasive biomarkers? (e.g., exhaled breath)
- Develop wearables for dust monitoring for workers and in the workplace
- Assess the rate of health monitoring in at risk industries
- What are the barriers to implementing currently known dust control measures?
- What are the most effective harm minimisation risk reduction strategies for preventing silicosis in workers across the diversity of workplaces?
- What are the barriers to implementing compliance measures/regulation /control measures/safe practices – moving beyond anecdotal evidence to empirical evidence?
- Can we develop technology that easily provides real time exposure monitoring for workers (personal devices)?

Other Research Questions

- What is the difference between relative risk across industries?
- What defines a worker at high risk (in each industry)?
- Risk stratification process – physical 'doing' of the task and what the person is (individual characteristics)
- How do we accurately find out what a person's exposure is (individual medical records) – national exposure registry?
- What are the barriers and systems to policy change which enable elimination of engineered stone?
- What are the factors to put in place to enable elimination? (think about other countries)
- How do we better communicate exposure levels and risk to workers? (government/industry)
- What are the barriers to a safe workplace?
- Engineering controls – design and evaluate dust monitoring technologies/solutions and evaluating these? E.g., how much water do we use?
- Is our compliance and enforcement system suitably robust to ensure proper compliance?
- Is it more cost effective to pour resources into screening than compliance?

- Decision support tools that are artificial intelligence- based for how an individual sole trader should implement controls and are they effective? [education space]
- Solution based / innovative solutions around prevention of exposure
- What are the barriers and enablers to improving and maximising workers understanding of their risk and how to minimise their risk?
- Would real time personal monitoring of different dust fractions serve to improve outcomes?
- What are the barriers to understanding and preventing silica exposure?
- What is the effect of total dust levels compared with respiratory silica in producing lung disease?
- Thoracic fraction of silica dust in monitoring equipment
- What makes an effective early warning system?
- Finding reliable measures to measure low levels of silica
- Can we accurately measure real-time silica exposure?
- What is the role of real time exposure monitoring in good exposure control?
- What can Australia learn from international approach to silica control?
- Understanding the interactions and effects of silica combined with other dusts
- What is the best way to provide the information to maximise compliance?
- What is the exposure profile of the national Australian workforce and community?
- Can available data be made available at a national level to inform future research?
- Who else is not being reached by current education and awareness?
- Can we assess the gap of knowledge in occupational hygiene across diversity of workplaces and groups?
- What is the cost benefit of prevention – both society and individual?
- How can technology support better on-site controls and prevention?
- Determine exposure thresholds
- How did existing compliance and regulations fail us?
- How can we better control silica dust in the workplace?
- Safe by design – how can the Hierarchy of Controls be applied to improve safety in a way that workers?
- What are the barriers and solutions to reducing silica exposure (applying the hierarchy of controls)?
- What is a silica safe workplace?
- What are the barriers to implementing the hierarchy of controls?
    - Across different workplaces?
    - What are the solutions (follow up)?
- What are the levels of secondary exposure by families/other, and how can this shape policy and practice?
- What are the health impacts of secondary exposure?
- What is the pathway/mechanism of health impacts from secondary exposure?
- What is the role and feasibility/cost-effectiveness of a ban of engineered stone?
- How can we effectively measure very low levels of respirable crystalline silica?
- How can we implement real time measurement of respirable crystalline silicaS?
- Can we determine a safe level of exposure?
- What is the risk of silicosis across industry/job role/other?
- What is the burden of silica exposure on the health system/society?
- What is the true financial cost of silicosis on the health system/country/individual?
- What is the cost-effectiveness of preventing silicosis compared to treating it?
- What are the co-factors which increase the risk of silicosis for people exposed to silica?
- What is the safety of alternative products, and the monitoring of historic products?
- What is the burden of silicosis from other industries?
- What are the co-factors associated with developing silica related disease?
- How can we improve the awareness of silica exposure?
- What is the current level of understanding of silica exposure/silicosis risk across industries?
- What are the most appropriate pre-clinical models for silicosis?

- Are we measuring the right thing? (size fracture, content of dust, etc.)
- Can we invent better, usable exposure monitoring technologies (need something faster)?
- Can we use data from silicosis diagnosis (lavage) to retrospectively understand characteristics of dust leading to silicosis?
- What are the demographics of high-risk cohorts? Sex, ethnicity etc. (retrospective?)
- What is the level of awareness in general practitioners of silicosis/importance of occupational history?
- What are the enablers/barriers for general practitioners in assessing and referring for silicosis diagnosis?
- What are the enablers/barriers of the workforce in awareness of silicosis?
- What are/were the enablers/barriers to implementation of the national registry?
- What can overcome barriers to implementing currently known dust control measures?
- Use of artificial intelligence technology in imaging to diagnose and predict progression
- Effectiveness of automated technologies/engineering controls in controlling dust exposure
- Implementation of educational modules for Hierarchy of Control compliance
- What are the risk factors for developing silicosis – personal and exposure related?
- Is there a safe level of exposure?
- What do we know about the toxicology of silica containing materials and their impact on risk?
- What are the barriers to compliance adherence?
- How do we motivate (incentivise) employers to want to be compliance – what are the factors to focus on?
- How do we create industry partnerships and secure industry involvement in reducing risk – i.e., equipment manufacturers so they see an economic and competition benefit?
- What is the feasibility/economic impact of eliminating engineered stone?
- What are the processes to risk assess new materials in construction/manufacturing?
- What is the optimal system to risk assess new materials in construction and manufacturing?
- What are the barriers to implementing the hierarchy of controls?

Priority Area: Screening and diagnosis

Top Research Questions

- What are the suitability and effectiveness of biological indicators?
- What is the appropriate risk informed optimal screening intervals?
- What are the approaches to and benefits of early testing and diagnosis?
- What are the biological indicators of exposure and risk to silica dust, including the development of novel technologies?
- How can occupational respiratory screening be harmonised with the current national lung cancer screening roll-out?
- What is the cost effectiveness of prevention, screening, and early diagnosis (both societal and health system)?
- What is the best model of screening practice (including risk prediction, screening intervals, cost-effectiveness, reporting, threshold for high resolution CT, other)?
- How can we optimise national data collection on:
    - Exposure
    - Screening
    - Diagnosis
- How can we identify novel diagnostic and prognostic biomarkers of silica-related disease?

Other Research Questions

- What workforce do we need in place to ensure adequate monitoring and screening?
- What are the optimal screening intervals pre-diagnosis? How much is enough?
- Risk analysis tool? Which screening tool should be used?
- Can we use artificial intelligence to diagnose silicosis? (international)

- Do we need to define minimum data sets (registry)?
- What are the better ways to sustain ongoing health monitoring?
- What is the research to ensure employers are maintaining ongoing health monitoring?
- Why do employers not pay for ongoing health monitoring?
- What proportion of people are screened?
- What are diagnostic techniques for use in remote locations?
- What are biological indicators of risk to silica exposure?
- What are the effective screening intervals for personalised risk?
- What is the role of breathomics?
- Comparison and understanding the difference of effectiveness of CT scans and X-rays?
- How can we use artificial intelligence to determine personalised risk and risk profiles?
- How can we develop accurate risk profiles to guide screening?
- What can we use for screening and diagnostics that is not invasive and can be used for diagnosis?
- How can we understand the burden of silica related lung cancer and other lung diseases?
- What is the role of lung cancer screening in people exposed to silica?
- How can we improve the current and extend this to post employment sector?
- How can the national registry be made as effective as possible?
- Is there something to be learnt from comorbidity and silica exposure?
- What would be required for the existing workforce to implement effective screening?
- How do we assess the economic burden of silica related disease?
- How do you better understand the causal link between occupational and non-occupational causes of disease?
- What are the ethical implications of screening?
- What are the mental health impacts of screening?
- How can silica exposure/a risk prediction tool be incorporated into the national lung cancer screening program?
- What is the optimal screening interval across industries? Or risk profile?
- What is the Optimal Care Pathway for silicosis?
- What is the best model of screening practice?
- What is the cost-effectiveness of screening?
- What data needs to be added to National Occupational Lung Disease Registry?
- What are the barriers to an effective national silica related diseases screening program?
- How can we upskill health professionals?

Priority Area: Treatment of silicosis

Top Research Questions

- How does silica exposure cause systemic autoimmune diseases?
- What is the pathogenesis of silicosis?
- What is the potential for new antifibrotics?
- Identify biomarkers (including genomics) for screening, early diagnosis, risk of progression and develop treatment pathways
- Identify best experimental models to recapitulate silicosis in humans and test therapies
- Determine effective measures of impairment and endpoints for treatment, including patient-centred outcomes

Other Research Questions

- What is the prevalence of mental health conditions in silicosis?
- What is the prevalence of autoimmune conditions in silica dust exposure?
- What are the biomarkers of early disease / exposure and of progressive disease? Are they different, why?
- Is there a biomarker which indicates progression?

- What are the genetic factors for lung fibrosis (general) compared to progressive massive fibrosis (silicosis)?
- What is the feasibility for establishing a national source of information sharing/generating in silicosis?
- Who is most likely to benefit from whole lung lavage?
- Are there sensitive/specific biomarkers in exhaled breath of disease?
- Identify genetic risk factors for people more likely to develop silicosis and also progressive disease
- Identify the link between silica exposure and autoimmune conditions
- Identify treatable traits for silicosis and determine optimal pathways
- Develop effective measure of impairment in silicosis for assessing compensation

Priority Area: Living with and managing the impact of silicosis

Top Research Questions

- What factors impact/influence mental health determinants and how are they related?
- What are the levers for change in improving the pathway and outcomes to compensation?
    - How do we identify the barriers?
- What is the best holistic/integrated/multidisciplinary optimal care model that is strongly linked to the consumer journey (not just medical model)?

Other Research Questions

- Scoping to understand the impact of silicosis across the spectrum
- What is the impact of silicosis on quality of life?
- How important is early return to work on mental health and wellbeing (if controls are in place)?
- How do we keep people in the industry they love?
- What strategies and programs can facilitate a safe return to work including in same/similar industry?
- What are impacts of not returning to work versus returning to work?
- What are the components of an optimal care pathway and how do we ensure accessibility?
- What are the facilitators and barriers?
- What are the experiences of people with silicosis who are seeking compensation – how does this differ in different jurisdictions?
- What can improve access to compensation for people living with dust diseases (taking a national approach)?
- What does an optimal supportive care model include for people with dust diseases?
- How important is care coordination and case management?
- How do we best ensure continuity of care?
- What is the effectiveness and cost effectiveness of an optimal care model?
- What are the factors that impact on equity of care provision and how do we address them?

REPRISE Checklist*

Note: The page numbers in this checklist refer to the submitted manuscript, not to the published article or its Supporting Information file

No	Item	Descriptor and/or examples	Page
A		Context and scope	
1	Define geographical scope	National – Australian states and territories	P5
2	Define health area, field, focus	Silicosis – identifying research priorities withing the areas of prevention, screening and diagnosis, treatment, and living with and managing the impacts of silicosis	P5
3	Define the intended beneficiaries	At risk workers, patients with silicosis	P5-6
4	Define the target audience of the priorities	Policy makers, funders, researchers, industry	P5
5	Identify the research area	Public health, health services research, clinical research, basic science	P5
6	Identify the type of research questions	Aetiology, diagnosis, prevention, treatment (interventions), prognosis, health services, psychosocial, behavioural and social science, economic evaluation, implementation	P8
7	Define the time frame	Long-term priorities	P5
B		Governance and team	
8	Describe the selection and structure of the leadership and management team	Lung Foundation Australia, Occupational Lung Disease Network Steering Committee	P7
9	Describe the characteristics of the team	Stakeholders from multiple disciplines	P7
10	Describe any training or experience relevant to conducting priority setting	Collaborated and sought advice from consultants experienced in priority setting	P7
C		Framework for priority setting	
11	State the framework used (if any)	Modified James Lind Alliance framework	P5
D		Stakeholders or participants	
12	Define the inclusion criteria for stakeholders involved in priority-setting	Patients, caregivers, at-risk workers, health professionals, researchers, policy makers, non-governmental organisations, government, industry	P6-7
13	State the strategy or method for identifying and engaging stakeholders	Partnership with organisations, social media, recruitment through hospitals	P7-8
14	Indicate the number of participants and/or organizations involved	164 participants were involved	P9, Table S2
15	Describe the characteristics of stakeholders	Healthcare professionals including occupational and respiratory physicians, allied health including nurses, physiotherapists, occupational hygienists, social workers, researchers including basic scientists, epidemiologists, implementation science experts, people with silicosis, at-risk workers, caregivers, industry representatives.	P6-8

No	Item	Descriptor and/or examples	Page
16	State if reimbursement for participation was provided	Travel and accommodation provided to the ONSC members from interstate	-
E		Identification and collection of research priorities	
17	Describe methods for collecting initial priorities	Online anonymous survey, face to face and virtual workshops, using nominal group technique	P6-8
18	Describe methods for collating and categorizing priorities	Thematic analysis	P7
19	Describe methods and reasons for modifying (removing, adding, reframing) priorities	Based on scope, clarity, definition, duplication, after feedback from research forums	P7-8
20	Describe methods for refining or translating priorities into research topics or questions	Reviewed by Steering Committee and project team	P7-8
21	Describe methods for checking whether research questions or topics have been answered	To be undertaken	n/a
22	Describe number of research questions or topics	Four pre-specified research priority areas, 5-9 priority topics per area, 3 top research questions per area, 12-60 total research questions per area	P9
F		Prioritisation of research topics/questions	
23	Describe methods and criteria for prioritizing research topics or questions	Nominal group technique	P8-9
24	State the method or threshold for excluding research topics/questions	Thresholds for ranking scores	P8
G		Output	
25	State the approach to formulating the research priorities	Via priority areas	P7-8
H		Evaluation and feedback	
26	Describe how the process of prioritization was evaluated	Feedback surveys were undertaken following the research forums, feedback was undertaken by the Steering Committee at the end of the process	-
27	Describe how priorities were fed back to stakeholders and/or to the public; and how feedback (if received) was addressed and integrated	Report delivered to Australian Government as requested, published manuscript, presentation at National conferences, online via LFA	-
I		Implementation	
28	Outline the strategy or action plans for implementing priorities	Communication with target audience, via policies and funding	P23

No	Item	Descriptor and/or examples	Page
29	Describe plans, strategies, or suggestions to evaluate impact	Integration in decision-making, funding allocation, review of relevant documents, in five years	P22
J		Funding and conflict of interest	
30	State sources of funding	Funded by the Australian Government	P24
31	Declare any conflicts or competing interests	As outlined in author conflict of interest statements	P24

* Tong A, Synnot A, Crowe S, et al. Reporting guideline for priority setting of health research (REPRISE). BMC Med Res Methodol 2019; 19: 243.
